# Fatal case of subdural empyema caused by *Campylobacter rectus* and *Slackia exigua*

**DOI:** 10.4322/acr.2023.433

**Published:** 2023-05-24

**Authors:** Yuki Munekata, Saki Yamamoto, Shun Kato, Yutaro Kitagawa, Ken Enda, Nanase Okazaki, Satoshi Tanikawa, Zen-ichi Tanei, Yohei Ikebe, Takahiro Osawa, Soichiro Takamiya, Hideki Ujiie, Masahiro Onozawa, Satoshi Hirano, Miki Fujimura, Shinya Tanaka

**Affiliations:** 1 Hokkaido University Hospital, Clinical Training Center, Sapporo, Japan; 2 Hokkaido University Hospital Department of Surgical Pathology, Sapporo, Japan; 3 Hokkaido University, Institute of Chemical Reaction Design and Development (WPI-ICReDD), Sapporo, Japan; 4 Hokkaido University, Faculty of Medicine, Department of Cancer Pathology, Sapporo, Japan; 5 Hokkaido University, Faculty of Medicine, Center for Cause of Death Investigation, Sapporo, Japan; 6 Hokkaido University Hospital, Department of Renal and Genitourinary Surgery, Sapporo, Japan; 7 Hokkaido University Hospital, Department of Neurosurgery, Sapporo, Japan

**Keywords:** Campylobacter rectus, Empyema, Subdural, Hematoma, Subdural, Sinusitis

## Abstract

We report a fatal subdural empyema caused by *Campylobacter rectus* in a 66-year-old female who developed acute onset of confusion, dysarthria, and paresis in her left extremities. A CT scan showed hypodensity in a crescentic formation with a mild mid-line shift. She had a bruise on her forehead caused by a fall several days before admission, which initially raised subdural hematoma (SDH) diagnosis, and a burr hole procedure was planned. However, her condition deteriorated on the admission night, and she died before dawn. An autopsy revealed that she had subdural empyema (SDE) caused by *Campylobacter rectus* and *Slackia exigua*. Both microorganisms are oral microorganisms that rarely cause extra-oral infection. In our case, head trauma caused a skull bone fracture, and sinus infection might have expanded to the subdural space causing SDE. CT/MRI findings were not typical for either SDH or SDE. Early recognition of subdural empyema and prompt initiation of treatment with antibiotics and surgical drainage is essential for cases of SDE. We present our case and a review of four reported cases.

## INTRODUCTION

Subdural empyema (SDE) is an infection between the dura and the arachnoid membranes.^1-3^ SDE commonly affects young and middle-aged people with a male predilection.^1,3-6^ Regarding its etiology, meningitis is the most common cause of SDE in infants. The sources of SDE are otitis media and sinus infection in older children and adults.^1,7^ Without immediate and appropriate management, SDE can be fatal, and the mortality rate has been reported to be between 6% and 35%.^8^ Moreover, SDE causes long-term complications such as hydrocephalus, residual hemiparesis, and epilepsy in more than half of the cases.^9^ Computed tomography (CT) and magnetic resonance imaging (MRI) have been the gold-standard methods for the diagnosis of SDE.^10-12^ However, prompt and accurate diagnosis of SDE is often tricky since its imaging results may resemble subdural hematoma (SDH).^1,4,6,7^ In this report, we present a fatal case of *Campylobacter rectus*-induced subdural empyema mimicking SDH that showed atypical characteristics on imaging tests confirmed by autopsy.

## CASE REPORT

A 66-year-old female developed acute onset of confusion, dysarthria, and paresis in her left extremities during Axitinib treatment started 1 month earlier as a fourth-line treatment for metastatic renal cell carcinoma. The next day, she visited the urology doctor in charge and was referred to our neurosurgery department. Her vital signs were not remarkable. Glasgow Coma Scale was E2V5M6. Neurological examination on admission revealed dysarthria, left-sided sensory disturbance, and motor hemiparesis. Manual muscle test grades of both upper and lower left extremities were 3 out of 0 to 5, while the right limbs scored 5.

Laboratory data showed an increased white blood cell (WBC) count of 39,000 /µL (normal range 3,300-8,600 /µL) and significantly elevated C-reactive protein (CRP) of 18.88 mg/dL (normal range 0-0.14 mg/dL). The levels of CRP remained high at nearly 10 mg/dL during the clinical course of refractory renal cell carcinoma. In contrast, the levels of WBC had been within the normal range until the previous visit, which was 17 days before admission. The remaining laboratory exams showed hypoalbuminemia, hypokalemia, and mild increases in the creatinine level.

**Figure 1:**
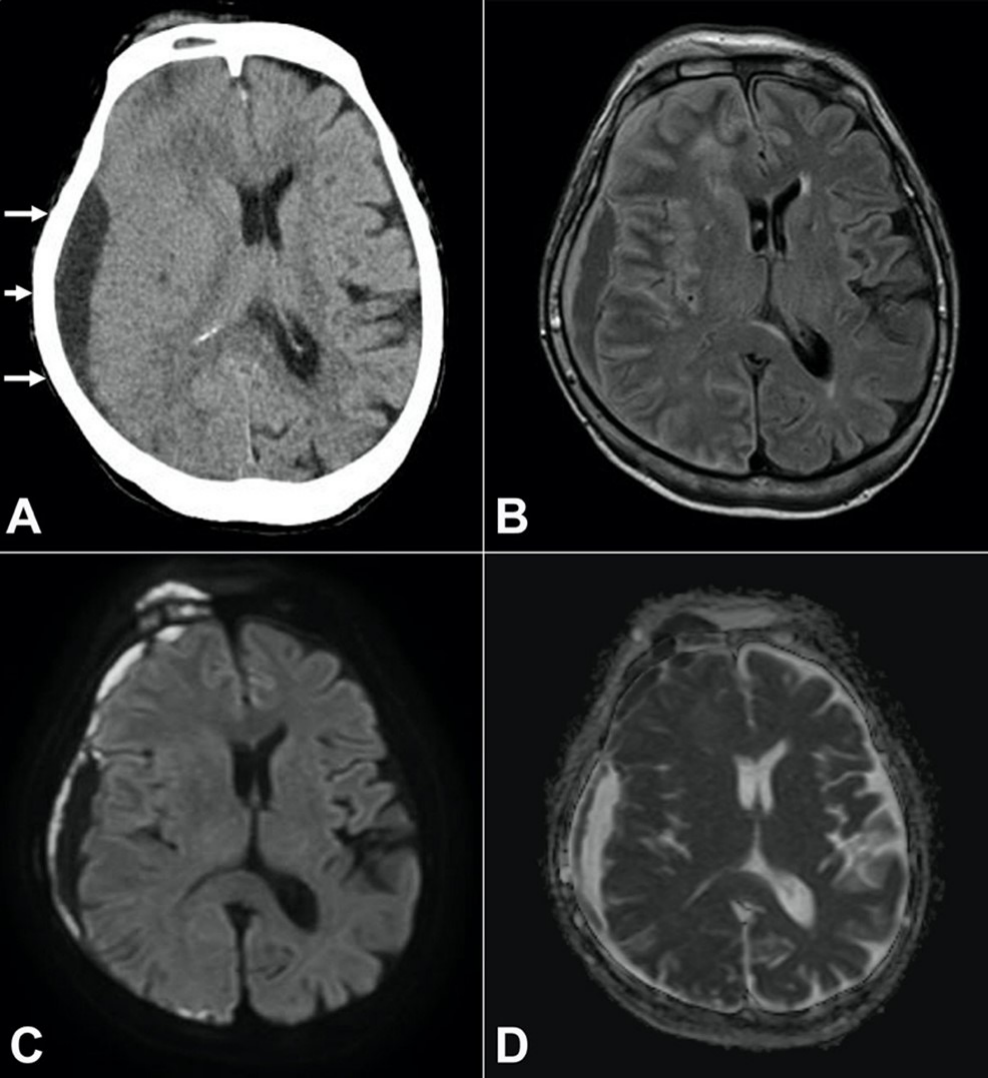
Head CT (**A**) and MRI (**B-D**). **A -** A CT scan showed a hypodense crescentic collection in the subdural space (arrow) with a mild mid-line shift. A swollen mass was also seen on the right frontal head; **B -** A FLAIR image showed low signal intensity in a crescentic formation surrounded by a peripheral hyperintense rim; **C -** DWI showed that the crescentic cavity consisted of internal hypointensity and linear hyperintensity of the dura mater extended to the right frontal contusion site. The subcutaneous mass on the right frontal head, an adjacent frontal bone, and lobe also showed a restrictive pattern, most likely indicating a subdural hematoma accompanied by bone and brain contusions; **D -** The ADC value of the central area of the crescentic collection was high, whereas its peripheral region showed a low ADC value.

A non-contrast brain CT scan revealed a subdural hypodense collection in a crescentic formation along with the right convexity and mild mid-line shift toward the left side (Figure 1A). A subcutaneous swollen mass accompanied the lesion on the right frontal head with a hyperdense core. Moreover, mucosal thickening and fluid accumulation were observed in the frontal sinuses. MRI showed that the crescentic region had low signal intensity surrounded by a peripheral hyperintense rim on fluid-attenuated inversion recovery (FLAIR) images (Figure 1B). The diffusion-weighted imaging (DWI) showed an internal hypointensity cavity associated with surrounding hyperintensity signals similar to the FLAIR sequence images (Figure 1C). On the corresponding apparent diffusion coefficient (ADC) map, the central area of the crescentic collection had a high value, in contrast to the low value of the peripheral region (Figure 1D). Regarding the swollen subcutaneous lesion on the right frontal head, the lesion had hypo- and hyperintense signals on T1- and T2-weighted images, respectively. The right frontal bone and frontal lobe just below the swollen mass also had hyperintensity on DWI with a low ADC value (Figure 1C, D).

Detailed history taken from a family member revealed the episode of an accidental falling with head trauma several days before admission. The fall was thought to have been due to left-sided hemiparesis caused by a chronic subdural hematoma. Chronic subdural hematoma with traumatic subcutaneous hemorrhage was suspected. Therefore, she was admitted to our neurosurgery ward, and a burr hole drainage was planned.

However, the patient’s condition rapidly deteriorated into pulseless electrical activity (PEA) at night, and she died despite long-term resuscitation efforts. A CT scan performed after resuscitation showed no notable imaging change lacking evidence of massive bleeding or brain stem herniation. In an attempt to reveal the cause of her death, an autopsy was performed.

## AUTOPSY FINDINGS

**Figure 2:**
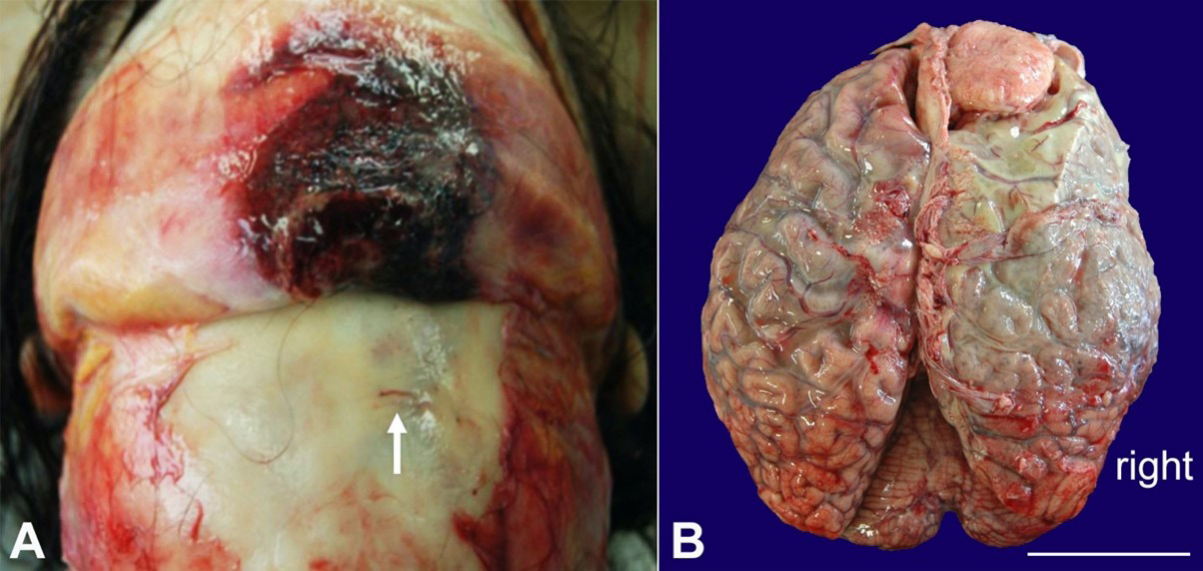
Macroscopic findings of the skull and brain. **A -** Hemorrhage under the bruise was evident on reversed skin, and a small bone fracture was found on the surface of the skull (arrow); **B -** Cerebral sulci were obscure on the right frontoparietal lobe due to subarachnoid pus collection that partly spread to the left convexity. Extra-axial meningioma measuring 45 mm in diameter, occupied the right frontal region, which was periodically followed without surgical intervention (scale bar= 5 cm).

A bruise on her right forehead, covering a subcutaneous hemorrhage, and a tiny fracture of the frontal skull were seen (Figure 2A). At the opening of the skull, the crescentic formation represented a copious amount of purulent secretion. The gross examination of the brain revealed a right-sided abscess within the subdural and subarachnoid spaces without evidence of bleeding (Figure 2B).

**Figure 3:**
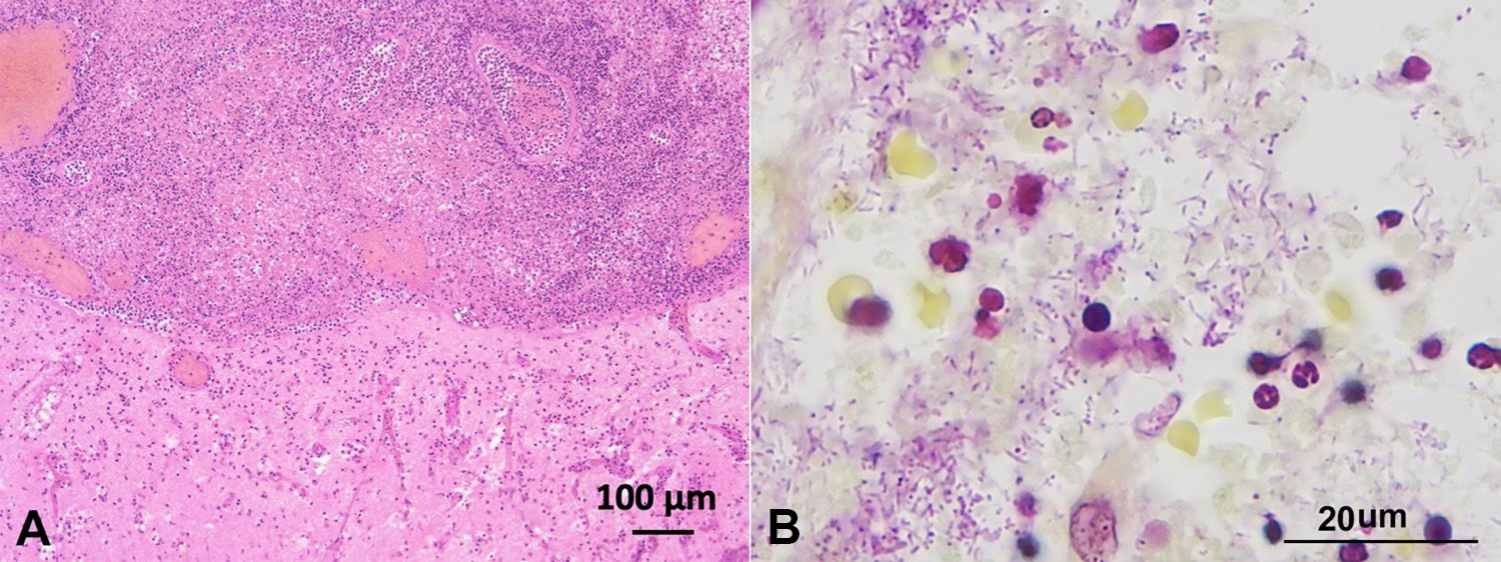
Photomicrographs of the brain (**A**) and purulent fluid (**B**). **A -** An abscess filled the subdural and subarachnoid space. Neutrophils extended to the brain parenchyma associated with cerebritis (lower half) (H&E stain); **B -** Two distinct types of microorganisms, Gram-negative rods and Gram-positive coccobacillus were observed in the purulent material (Gram stain).

On microscopy, the abscess partly invaded the brain parenchyma, which was surrounded by mild cortical microvacuolation exhibiting edema (Figure 3A). Neutrophils with numerous gram-negative rods and concomitant Gram-positive coccobacillus were observed in the purulent material (Figure 3B).

Evidence of severe inflammation underpinned by massive neutrophil invasion was seen in other organs, including the liver and spleen. In addition, the patient’s cerebrospinal fluid (CSF) culture bottles yielded *Campylobacter rectus* and *Slackia exigua*. Thus, we concluded that the patient’s death had been caused by sepsis secondary to meningoencephalitis derived from subdural empyema. Regarding the cause of subdural empyema, *Campylobacter rectus,* and *Slackia exigua* were the most likely causative microorganisms based on the autopsy findings.

## DISCUSSION

Herein, we describe septic meningoencephalitis secondary to a *Campylobacter rectus* and *Slackia exigua* subdural empyema. SDE’s most common etiological microorganisms are anaerobes, aerobic *Streptococci, Staphylococci, Haemophilus influenzae, Streptococcus pneumoniae*, and other gram-negative bacilli.^13^ In subdural empyema secondary to paranasal sinusitis cases, the anaerobic and microaerophilic streptococci such as *Streptococcus milleri* and *Streptococcus anginosus* are the most common reported microorganisms.^1,9,13,14^ *Campylobacter rectus* detected in our case’s CSF culture is a rare SDE pathogen. *C. rectus*, previously known as *Wolinella recta*, is an anaerobic gram-negative rod comprising the normal oral subgingival flora. The association between periodontal disease and this *Campylobacter* species is well known.^15^ In contrast, extraoral infection by *C. rectus* was reported in only a few cases in the literature. Up to our literature search, only four cases of subdural empyema due to *C. rectus* have been reported (Table 1).^15-18^

**Table 1:** Reported cases of subdural empyema caused by *Campylobacter rectus* infection

**Ref**	**Age/ Sex**	**Symptoms**	**WBC (/μL)**	**CRP (mg/dL)**	**Comorbidity**	**Suspicious source of infection**	**Location**	**Surgery**	**Antibiotics**	**Outcome**
**^15^**	54/M	headache, mental status changes, fever, forgetfulness, inappropriate behaviors, dysarthric, nasal mucus, fever	23,400	-	DM, HT, HL, smoking	sinusitis	left anterior hemisphere	D	VCM, CAZ, MTZ, CTRX, PCG	alive
**^16^**	41/F	headache, ptosis, diplopia, palsy, fever	13,000	4.36	DM	ruptured mycotic aneurysm	interhemispheric fissure, supratentorial regions	D	VCM, CTRX, MTZ	dead
**^17^**	66/M	vertigo, gait instability, wight loss	15,500	2.58		tooth abscess, otitis media	multiple abscesses	D	MEPM, DOX	alive
**^18^**	79/F	headache, facial pain, fall at home, vomiting, right lower limb paresis, dehydration	-	-		dental abscess, sinusitis	frontotemporal abscess	D	CPFX, MEPM	alive
**Our case**	66/F	palsy, discover of consciousness, deviation, muscle weakness, hypoesthesia	39,000	18.88	renal cell carcinoma, HT, smoking	sinusitis	right anterior and temporal hemisphere	-	-	dead

Abbreviations: M, male; F, female; D, drainage; DM, diabetes mellitus; HT, hypertension; HL, hyperlipidemia; VCM, vancomycin; CAZ, ceftazidime; MTZ, metronidazole; CTRX, ceftriaxone; PCG, penicillin G; MEPM, meropenem; DOX, doxycycline; CPFX, ciprofloxacin.

Three of these 4 cases had a dental abscess and/or sinusitis. Our case had sinusitis, which was pointed out in the MRI study conducted 10 months before hospitalization. The sinusitis was not treated and had been expanded from the maxillary sinus to the frontal sinus. Increased WBC and CRP were thought to indicate a bacterial infection in those case reports. In our case, the high levels of CRP for several months before the onset of the neurologic symptoms were considered due to advanced-stage renal cell carcinoma. Another microorganism, *Slackia exigua*, is a Gram-positive, obligate anaerobic coccobacillus associated with dental infection but rarely causes extraoral disease.^19^ To the best of our knowledge, there have been no reports of SDE caused by *Slackia exigua*.

Another unique point of our case is its MRI presentation. MRI is superior to CT in demonstrating extra-axial fluid and rim enhancement, and DWI is particularly helpful in differentiating SDE and SDH.^1,10^ SDE is characterized by hyperintensity on DWI with a low apparent diffusion coefficient (ADC) value, indicating restricted diffusion^1,9,10^. This restriction is thought to be partially due to the viscosity of the empyema fluid^1,10^. In our case, however, there were uncommon findings of hypointense signals encompassed by a restrictive capsule on DWI with a reversed pattern on an ADC map. The atypical findings might be due to the low empyema fluid viscosity showing restricted diffusion since the development of the infection was considerably rapid.

Management of SDE mainly includes early initiation of antibiotic therapy and surgical procedures, though SDE is occasionally cured with antibiotic treatment alone. Hence, early surgical intervention by burr hole drainage or craniotomy evacuation is the key to diagnosing and treating SDE, and administration of adequate antibiotics might result in timely recovery and salvage of maximal neurological function.^7,9,15-18^

## CONCLUSION

We experienced a fulminant subdural empyema caused by coinfection of uncommon etiologic agents, *Campylobacter rectus* and *Slackia exigua*, manifesting unusual imaging traits. Early diagnosis of SDE and administration of adequate antibiotics are essential for managing the dreaded complication.
